# Double Transseptal Puncture for Catheter Ablation of Atrial Fibrillation: Safety of the Technique and Its Use in the Outpatient Setting

**DOI:** 10.4061/2010/295297

**Published:** 2010-12-13

**Authors:** Laurent M. Haegeli, Thomas Wolber, Ercüment Ercin, Lukas Altwegg, Nazmi Krasniqi, Paul G. Novak, Laurence D. Sterns, Corinna B. Brunckhorst, Thomas F. Lüscher, Richard A. Leather, Firat Duru

**Affiliations:** ^1^Department of Cardiology, Royal Jubilee Hospital, Victoria, BC, Canada V8R1J8; ^2^Cardiology, Cardiovascular Center, University Hospital Zurich, Rämistrasse 100, 8091 Zurich, Switzerland

## Abstract

*Introduction*. For pulmonary vein isolation in patients with atrial fibrillation (AF), some centers use the double transseptal puncture technique for catheter access in order to facilitate catheter manipulation within the left atrium. However, no safety data has so far been published using this approach. *Method*. 269 ablation procedures were performed in 243 patients (mean age 56.6 ± 9.3 years, 75% men) using the double transseptal puncture for catheter access in all cases. Patients were considered for ablation of paroxysmal (80%), persistent (19%), and permanent (1%) AF. 230 procedures were performed on an outpatient basis (85.5%), and 26 were repeat procedures (9.7%). *Results*. The double transseptal puncture catheter access was successfully achieved in all patients. The procedural success with the endpoint of pulmonary vein isolation was reached in 255 procedures (95%). A total of 1048 out of 1062 pulmonary veins (99%) were successfully isolated. Major complications occurred in eight patients (3.0%). Of these, seven patients (2.6%) had pericardial effusion requiring percutaneous drainage, and one patient (0.4%) suffered a minor reversible stroke. One patient (0.4%) had a minor air embolism with transient symptoms. *Conclusion*. The double transseptal puncture catheterization technique allows easy catheter manipulation within the left atrium to reach the goal of acute procedural success in AF ablation. Procedure-related complications are rare, and the technique can be used safely for AF ablation in the outpatient setting.

## 1. Introduction

Atrial fibrillation (AF) is the most common of all cardiac arrhythmias with an incidence and prevalence on the rise. Radiofrequency catheter ablation is widely performed as an effective treatment for recurrent, drug-resistant AF [1–4]. Pulmonary vein isolation guided by circumferential mapping is the cornerstone in the catheter ablation for AF [5, 6]. The transseptal puncture is the conventional approach to access the left atrium, which was initially developed and described by Ross et al. for the measurement of left atrial pressure and for mitral valve repair [7]. Recently, the technique is routinely used in electrophysiologic laboratories in order to percutaneously map and ablate left atrial arrhythmias, especially AF [8]. 

When the electrophysiological or the combined electrical and anatomic approach for the ablation of AF aims for isolation of the pulmonary veins, two catheters have to be introduced by the transseptal access into the left atrium for mapping of the pulmonary veins and for radiofrequency ablation, respectively. This is generally achieved by the single-puncture, double transseptal catheterization technique as a second transseptal puncture can theoretically bear additional risks inherent to the transseptal puncture, such as perforation resulting in pericardial tamponade, coronary artery spasms provoked by mechanical irritation on the interatrial vagal network, air embolism, and thromboembolism [9]. Some centers use double transseptal puncture for catheter access in order to facilitate catheter manipulation within the left atrium and to avoid iatrogenic atrial septal defects [10]. Ablation techniques have improved and the complication rate has decreased, but no safety data has so far been published using the double transseptal puncture approach [11–15]. 

## 2. Methods

### 2.1. Study Patients

Consecutive patients with symptomatic paroxysmal and persistent AF undergoing a percutaneous catheter ablation procedure at the Royal Jubilee Hospital in Victoria BC (Canada) and at the University Hospital of Zurich (Switzerland) were included in this study. Demographic and clinical data were collected prior to the procedure. All patients gave informed consent prior to the procedure. Clinical results at followup including late complications were collected using a patient assessment form at 3 and 6 months after the intervention.

### 2.2. Double Transseptal Puncture

All patients underwent wide-area circumferential pulmonary vein isolation. Additional linear lesions for persistent AF were applied in the left atrium at the discretion of the operator. For that purpose, three femoral vein sheaths were inserted under local anesthesia. A steerable decapolar electrophysiology catheter was placed into the coronary sinus and a diagnostic quadripolar catheter at the His bundle. The two separate transseptal punctures were performed using a Brockenbrough transseptal needle (Type BRK N°1; St. Jude Medical Inc., St. Paul, MN, USA) and two 8F transseptal guiding introducers (Fast-Cath SL1; St. Jude Medical Inc., St. Paul, MN, USA). The first 8F transseptal guiding introducer was placed in the right femoral vein and advanced over a guidewire into the superior vena cava under fluoroscopic guidance. The guidewire was replaced with the Brockenbrough needle which was connected to a pressure curve line. Both Brockenbrough needle and transseptal guiding sheath were withdrawn in caudal direction keeping the needle and sheath hub arrow rotated between 4 and 5 o'clock position under fluoroscopic guidance in 8° left anterior oblique projection until the tip engaged the fossa ovalis. After the position was verified within the fossa ovalis by fluoroscopy in a 10° right anterior oblique projection, and a 15° left anterior oblique projection, just the needle was advanced across the septum. After left atrial pressure curve monitoring, aspiration of oxygenated blood and contrast dye injection confirmed the correct needle tip position within the left atrium, the transseptal assembly was advanced into the left atrium. For the second transseptal puncture, the His mapping catheter was replaced with a second 8F transseptal guiding introducer, which was inserted into the left atrium by the same way as the first transseptal puncture. 

**Figure 1:**
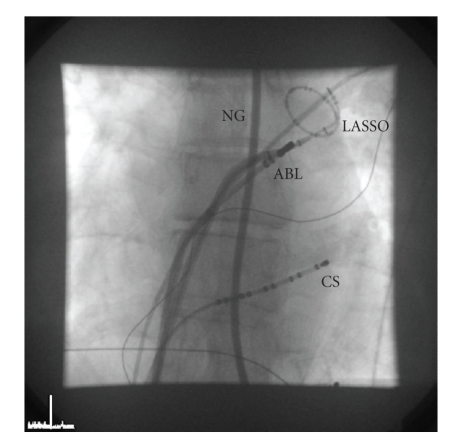
Fluoroscopy image in anterior posterior view. Both the ablation catheter and the circular mapping catheter are inserted into the left atrium and positioned at the ostium of the left superior pulmonary vein after successful double transseptal puncture of the interatrial septum. Esophageal position is monitored using a contrast dye-filled nasogastric tube. ABL, ablation catheter; LASSO, circular mapping catheter; CS, coronary sinus catheter; NG, nasogastric tube.

Immediately after having positioned both transseptal sheaths across the interatrial septum, therapeutic anticoagulation was initiated with a bolus of intravenous heparin (100 IU/kg). Activated clotting time (ACT) was monitored in 30-minute intervals followed by additional boluses of heparin to keep the ACT with a target of 300 seconds. During the transseptal catheterization, continuous monitoring of the electrocardiogram is performed to detect any coronary artery spasm provoked by the mechanical effects of the transseptal puncture on the interatrial vagal network or coronary artery embolism, and monitoring of the cardiac silhouette excursion on fluoroscopy and monitoring of the systemic blood pressure are performed to detect any impending pericardial tamponade. A circular mapping catheter and an ablation catheter were inserted through the transseptal sheaths for mapping and ablation at the pulmonary vein orifice (Figure 1).

### 2.3. Oral Anticoagulation Strategy

Warfarin was discontinued three days prior to the procedure to allow INR to drop in a subtherapeutic range (INR < 2.0) on the day of the procedure. Oral anticoagulation was resumed on the same day of the procedure for a duration of at least 3 months without bridging by low-molecular heparin.

### 2.4. Outpatient Setting for AF Ablation

The patients were admitted to the outpatient facility on the morning of the procedure and were brought to the electrophysiologic laboratory in a nonsedated state. The procedure was performed under conscious sedation using midazolam and fentanyl. After the sheaths were pulled out, the patients were monitored for at least five hours and discharged if they were stable on the same day. No routine echocardiogram was performed. The outpatient facility was staffed overnight and could accommodate outpatient patients if overnight observation and care were required [16]. 

## 3. Results

A total of 269 consecutive AF ablation procedures were performed in 243 patients. The mean age of the study population was 56.6 ± 9.3 years (75% male). The patient characteristics are shown in Table 1. Of the 269 ablation procedures, 230 were performed on an outpatient basis (85.5%), whereas the remaining 39 patients (14.5%) were kept overnight because of major complications (8 patients; 3%), or due to minor groin bleeding, late completion of the procedure, prolonged sedation, or reduced well-being of the patient. The analysis included 26 repeat ablation procedures (9.7%). The procedural characteristics are shown in Table 2. 

The double transseptal puncture techniques were performed for left atrial catheter access using two separate transseptal punctures through two separate transseptal holes, rather than using the same hole of the first transseptal puncture. One patient required additional transesophageal guidance for the transseptal punctures because of difficulty to cross the fibrotic interatrial septum due to prior procedures. In all cases, ablation catheter and circular mapping catheter were easily manipulated within the left atrium and the acute procedural success with the endpoint of complete isolation of all pulmonary veins was reached in 255 procedures (95%). A total of 1048 out of 1062 pulmonary veins (98.7%) were successfully isolated.

**Figure 2:**
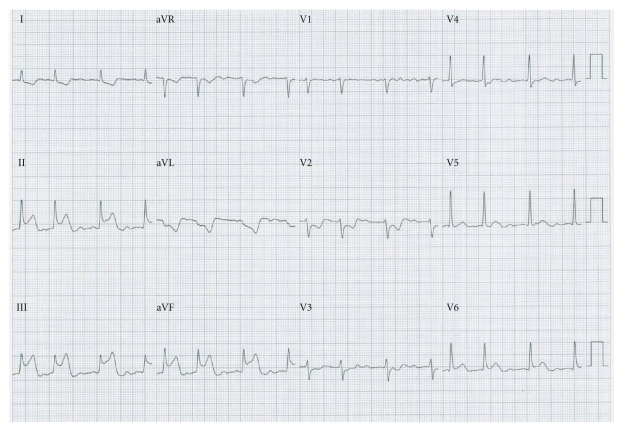
12-lead ECG of a patient with Bezold-Jarisch-like reflex. After passing the catheters through the transseptal sheaths, a transient ST elevation was observed in leads II, III, and aVF, which resolved spontaneously after 5 minutes.

Major complications occurred in 7 outpatients and one inpatient accounting for a combined incidence of 3.0%. Of these, 7 patients (2.6%) had pericardial tamponade, which was successfully drained percutaneously during or at the end of the intervention in all cases. No delayed pericardial tamponades were observed. One patient (0.4%) suffered a minor reversible stroke with manifestation of numbness in one hand. One patient (0.4%) had a minor reversible air embolism with transient symptoms after passing the sheaths through the interatrial septum. Another patient (0.4%) had a typical Bezold-Jarish-like reflex with documentation of a sudden and transient ST-segment elevation in the inferior leads, a phenomenon already reported as coronary artery spasm provoked by mechanical irritation by transseptal puncture of the interatrial vagal innervation (Figure 2) [17]. There were neither acute nor late peripheral or cerebral thromboembolic events. None of the procedures were complicated by vascular access complications requiring transfusion or surgical interventions.

## 4. Discussion

Catheter ablation of AF by pulmonary vein isolation can be performed using two transseptal catheters, one circular mapping catheter and one ablation catheter, which are inserted into the left atrium through either a single transseptal puncture technique or by using separate transseptal punctures. The transseptal puncture is a demanding procedure requiring specific expertise related to the concern of the possible occurrence of serious complications. Some centers perform the single-puncture, double transseptal catheterization to reduce the potential risk generated by a second transseptal puncture, and others choose the double transseptal puncture for catheter access in order to facilitate catheter manipulation within the left atrium. In this paper, we report the safety of the double transseptal puncture for catheter access in a large cohort of consecutive patients undergoing catheter ablation of AF. The technique could be successfully performed in all procedures, enabling easy catheter manipulation without the catheters “sticking” to each other, which might occur with the single-puncture, double transseptal catheterization technique. This may explain that the procedural endpoint of pulmonary vein isolation could be achieved for the vast majority (98.7%) of the ablated pulmonary veins in our patients. 

Catheter ablation on an outpatient basis, which is already established to be feasible and safe in the treatment of supraventricular tachyarrhythmias, was also feasible in the majority of patients in our cohort undergoing the double transseptal puncture technique for catheter ablation of AF. Same day discharge is an attractive model in light of increasing numbers of AF ablation procedures leading to logistic and health-economical constraints. Moreover, from a patient's perspective, outpatient procedure to early ambulation increased patient comfort and early return to work [18]. Our study showed that procedure-related complications are rare, and the technique can be used safely for AF ablation in the outpatient setting.

The incidence of major complications in our cohort was comparable to that reported in the scientific literature for AF ablation [19]. No acute major complication related to the transseptal punctures was reported. Pericardial effusion requiring percutaneous drainage (2.6%) and the single minor reversible cerebrovascular event (0.4%) manifested in all patients close to the end or soon after completion of the procedure, suggesting that these might be due to the ablation itself (longer procedure duration, multiple radiofrequency energy applications, and prolonged catheter manipulation), rather than the transseptal intervention while crossing the interatrial septum. 

Similar complication rates were reported by Fagundes et al. in a retrospective patient cohort using a single-puncture, double transseptal access for AF ablation [20]. In their cohort, no iatrogenic atrial septal defect was detected during followup. In contrast to this observation, the study of Hammerstingl et al. demonstrated a higher incidence of long-term persistent iatrogenic atrial septal defects after single transseptal puncture catheterization as compared to those that occur after the double transseptal puncture technique [10]. In 8 out of 27 patients who underwent the single transseptal puncture with additional advancement of a second catheter into the left atrium, an iatrogenic atrial septal defect persisted compared with no iatrogenic atrial septal defect in 15 patients using the double transseptal puncture technique. Traumatic dilatation of the single transseptal puncture by two catheters inserted into the left atrium might result in severe endothelial denudation promoting thrombosis and risk for thromboembolism as shown by intracardiac ultrasound observation in another study by Maleki et al. [21]. 

### 4.1. Study Limitations

This is an observational cohort study and is not a prospective, randomized trial. In the absence of a direct randomized comparison of the double transseptal puncture technique versus the single transseptal puncture access for the ablation of AF, limited conclusions regarding superiority of one technique over the other in terms of safety and procedural outcome can be made. In addition, the clinical outcome was assessed based on symptoms reported by the patients only, and therefore, the reliability of the presented data on outcome is limited due to known difficulties of survey-based studies.

## 5. Conclusion

The double transseptal puncture catheterization technique allows easy catheter manipulation within the left atrium to reach the goal of acute procedural success in AF ablation. Procedure-related complications are rare and the technique can be used for AF ablation in the outpatient setting. However, it needs to be investigated in randomised studies if the double transseptal puncture technique is superior to the single-puncture, double transseptal catheter access for AF ablation in terms of safety and procedural success.

## Figures and Tables

**Table 1:** 

Patient characteristics	*n* = 243
Age (years)	56.6 ± 9.3
Sex (male)	183 (75%)
Type of AF	
Paroxysmal AF	195 (80.3%)
Persistent AF	45 (18.5%)
Permanent AF	3 (1.2%)
Duration of AF (years)	7.4 ± 6.2
Left ventricular ejection fraction (%)	57.9 ± 7.1
Left atrial diameter (mm)	43.3 ± 5.4

**Table 2:** 

Procedural statistics	*n* = 269
Average procedure time (min)	201 ± 31
Fluoroscopy time (min)	41.3 ± 13.0
Major complications	
(i) Thromboembolic event and stroke	1 (0.4%)
(ii) Pericardial tamponade	7 (2.6%)
(iii) Major vascular access complication (requiring surgery or blood transfusion)	none
Minor complications	
(i) Reversible air embolism	1 (0.4%)
(ii) Bazold-Jarish-like reflex	1 (0.4%)
